# AMERICAN INDIAN FEMALE CAREGIVERS: STRESS AND RESILIENCE

**DOI:** 10.1093/geroni/igz038.3423

**Published:** 2019-11-08

**Authors:** Felina Cordova-Marks, Robin Harris, Nicolette Teufel-Shone, Beatrice Norton, Ann Mastergeorge, James Cunningham, Lynn Gerald

**Affiliations:** 1 University of Arizona, Tucson, United States; 2 Northern Arizona University, Flagstaff, United States; 3 Hopi Office of Aging and Adult Services, Kykotsmovi, United States; 4 Texas Tech University, Lubbock, United States

## Abstract

Background/Introduction: Increased stress has been found to be a part of the caregiving experience. However, how stress is handled is important. Resilience has been shown to decrease stress in non-caregivers. There is a lack of information about American Indian (AI) caregiver stress. In this study, we seek to investigate if resilience acts as a stress buffer in this population of AI female caregivers.Methods: The Hopi Adult Caregiver Survey was conducted in 2017 with 44 female Hopi caregivers. Resilience and stress scale questions were asked as well as variables potentially affecting these. Resilience as measured by the Connor Davidson Resilience Scale-10 (CD-RISC) and stress as measured by the Perceived Stress Scale-10 scores were calculated as well as categorical levels of higher and lower stress/resilience. Variables possibly associated with each were assessed using linear regression analyses. Results: Forty-four female caregivers were surveyed. The overall mean stress score for caregivers was 17.9 Â± 6.2 on the PSS. For difference between higher and lower stress, expectation of females to be caregivers, number of times using a traditional healer/traditional medicine person, number of caregiver difficulties, self-perceived health rating, self-perceived changes to eating habits since becoming a caregiver were significant. Average sum resilience score was 28.7 Â± 6.2 on the CD-RISC. In linear regression, it was found that with an increase in the resilience score, stress score decreased.Discussion: In these caregivers, resilience acts as a stress buffer. Increasing resilience and countering factors that decrease resilience may reduce stress experienced by caregivers.

